# Starting an undergraduate degree amid the COVID-19 pandemic: A mixed-method egocentric network study on student loneliness

**DOI:** 10.1371/journal.pone.0297953

**Published:** 2024-02-02

**Authors:** Leonie Richardson, Emily Long, Claire Goodfellow, Jelena Milicev, Maria Gardani

**Affiliations:** 1 Mental Health and Wellbeing, School of Health and Wellbeing, University of Glasgow, Glasgow, Scotland; 2 MRC / CSO Social and Public Health Sciences Unit, School of Health and Wellbeing, University of Glasgow, Glasgow, Scotland; 3 School of Health in Social Science, University of Edinburgh, Edinburgh, Scotland; Satyawati College (Eve.), University of Delhi, INDIA

## Abstract

Students who began their undergraduate university studies in the midst of the COVID-19 pandemic (the ‘COVID cohort’), may have been particularly at risk for experiencing increased loneliness. This study employed an exploratory egocentric network and mixed-methods approach to investigate the links between social networks and loneliness in the COVID cohort. Of sixty-one respondents meeting inclusion criteria for the study, fifty-eight first-year undergraduate students from the September 2020 intake at a large Scottish University provided egocentric network data via an online survey, as well as responses to three open-ended questions which were aimed at generating qualitative data about participants’ experiences of starting university in the context of the COVID-19 pandemic. Bivariate analyses suggest that having a larger social network, and higher satisfaction with that network, was associated with reduced loneliness. We additionally explored these associations in subsamples of students living on-campus and living off-campus. Our qualitative data adds valuable insight into the impact that pandemic-related social-distancing restrictions had on limiting students’ opportunities for meeting their peers and forging meaningful social connections at university. Limitations of this study include a small sample size and an exploratory approach requiring further investigation and replication. However, in the context of universities continuing to use hybrid teaching models, this study provides useful initial insights, highlighting potential avenues for institutions to support students in developing social connections in the transition to higher education.

## Introduction

Loneliness has been defined as an unpleasant and distressing emotional state, linked to the perception that one’s social needs are not met by the quantity or quality of one’s currently existing social relationships [1, 2]. A number of life events can lead to disruptions or changes in an individual’s social network [3] and support systems and can make them particularly vulnerable to experiencing feelings of loneliness [4, 5]. The transition to university is one such crucial time of change in young people’s lives [6]. Starting university increases exposure to a variety of stressors associated with adjusting to an entirely new academic and, importantly, social environment [7]. This period has indeed been found to coincide with increased feelings of loneliness among students [8], which has been associated with student anxiety and depression [9] and suggested to be an important factor in increasing the risk of university dropout [10, 11].

The university transition is often marked by changes in young people’s social networks, characterised by a process of increased independence and individuation from one’s family systems [12] and the progressive dissolution of previously established friendships and social and support networks [13]. Correspondingly, forming new social connections and friendships becomes a predominant concern for new undergraduate students [14] and is crucial to supporting young people’s socio-emotional adjustment to university [6, 15, 16]. Feelings of loneliness among first-year students have indeed been found to predict their adjustment to the new university environment [17] and this relationship appears to be mediated by perceived social connectedness and social support [18].

Examining first-year students’ social relationships and support networks is thus crucial to understanding student loneliness and is of particular importance given the negative and potentially pervasive consequences of loneliness for mental health, well-being and university adjustment.

### The COVID cohort

In the context of the COVID-19 pandemic, universities across the world and the UK were forced to suspend in-person classroom activities and pivot to remote, online teaching. This shift was implemented approximately from mid-March 2020 with intermittent disruption and with progressive return to hybrid and in-person teaching throughout the 2021/2022 academic year [19, 20]. For the autumn 2020 intake of new undergraduate students, the transition to university occurred under hitherto unprecedented circumstances, with notably reduced opportunities for in-person socialising, on-campus activity and social contact more generally [21].

Several studies have examined the impact of COVID-19-related restrictions on young people’s social lives and documented an increase in loneliness among students during the pandemic [22] Labrague and colleagues [23], on the other hand, identified perceived social support as a protective factor against student loneliness. Other studies have adopted a social network approach [24] to examine the impact of lockdowns on social networks during the pandemic, both among young people in general population [25] and student samples [26]. Social network analysis provides methods for systematically mapping, describing and characterising social networks as sets of relationships, ties or connections surrounding individuals [27]. Measuring social networks captures both quantitative (number of ties) and qualitative (e.g., perceived social support) information about individuals’ social relationships [28, 29] which may relate to loneliness. Elmer and colleagues [30], for instance, found that having a smaller social network was associated with students becoming lonelier in lockdown.

However, much of the literature focuses on students who had started their university degree prior to the outbreak of the pandemic and had thus previously experienced some level of teaching under pre-pandemic circumstances. The “COVID cohort” [31], however, is comprised of students who experienced their university transition in the context of social-distancing restrictions. As these students had less, if any, opportunities to meet university staff and peers and/or attend crucial induction events in-person [31] they may have been at an increased risk for loneliness.

Despite having been discussed as a group that may be particularly vulnerable to the negative consequences of the COVID-19 pandemic [32], limited studies have specifically sampled the COVID cohort. These studies have focused, for example, on students’ experience of online teaching methods [31] and demographic predictors of their overall mental health [33]. One study [22] focused on associations between university belonging, social connectedness, loneliness, mental health and well-being measures in a large sample (N = 1239) across three cohorts of first-year students (including the COVID cohort, the preceding and following cohorts). In this study, loneliness was found to be significantly higher in the COVID cohort compared to the other cohorts of students. Of note, in the overall sample, loneliness was linked to increased psychological distress and was negatively associated with university belonging and having social connections with multiple groups within and outside of university. However, these associations were not examined in the COVID cohort specifically.

Ultimately, given that starting first year at university amid the pandemic may indeed have put students at greater risk for loneliness, exploring students’ social relationships and their association with loneliness in the COVID cohort merits particular attention.

### The present study

The present study adopts an egocentric network approach [27] to explore associations between the characteristics of students’ social networks and loneliness in the ‘COVID cohort’. In egocentric network studies, participants (the ‘egos’) are systematically surveyed about the members of their social network (the ‘alters’). Egos are prompted to provide information about their relationships with and characteristics of each alter in their social network. The current study thus examined the size (the number of social ties an individual has) and composition (the types of relationships an individual has) of students’ ‘ego’ networks as well as their subjective perception of (the quality/supportiveness of) their relationships with alters. Additionally, we assessed students’ overall satisfaction with their ego networks.

Finally, to capture rich data on students’ experiences, the present study also included a qualitative component, aiming to enrich and corroborate our quantitative findings [34]. Using open-ended questions, we aimed to record first-year students’ own accounts of their experiences of starting university in the context of the COVID-19 pandemic, their experience of feelings of loneliness and the role played by their social networks in relation to their mental health and well-being. In sum, this study employs a mixed methods egocentric network approach to investigate the links between social networks and loneliness in the COVID cohort. To the best of the authors’ knowledge, this study is the first to report on associations between the characteristics of social networks and loneliness within the COVID cohort.

## Methods

### Study design and sample characteristics

Data were collected between November 16^th^ and December 21^st^, 2020, using a cross-sectional online survey accessed via an anonymous weblink to Qualtrics Survey Software (Qualtrics, Provo, UT). Study participants (N = 61) were first-year undergraduate students, aged 18 years or older, from the September 2020 intake at a large Scottish university, recruited via various social media channels (e.g., Facebook; Twitter) targeting newly enrolled first-year students. Participants were offered the opportunity to enter a raffle to win vouchers and/or to get course credits where applicable for their participation.

### Ethics

Ethical approval was obtained from authors’ institution (Ref: 300200075). Participants provided online consent to take part in the study via a Qualtrics form which preceded access to the main online study survey. Participants were only able to access the main study survey page if they selected the option indicating they consented to taking part in the study on the online consent form. If they selected the option to decline providing consent, or left the online field blank, they were redirected to a page thanking them for their response and indicating they could close the browser window. Survey data was anonymised for data analysis using unique numerical identifiers.

### Measures

#### Demographic variables

Participants’ demographic information as well as data on their living situation (living ‘on-campus’ in halls of residence; or living ‘off-campus’ at home or in an owned or rented accommodations), were collected.

#### Loneliness

The 3-item Loneliness Scale (UCLA-3) [35] consists of 3 statements asking how often respondents feel (1) they lack companionship; (2) left out; (3) isolated from others. Responses were on a scale from 1 (*Hardly ever*) to 3 (*Often*). Scores range from 3 to 9, with the top quintile (6–9) identifying high levels of loneliness [cf. 36].

#### Egocentric network characteristics

Following an egocentric network approach [27, 37] participants were first prompted to use pseudonyms to list up to 30 people (‘alters’) that were important in their lives. The total number of alters represented network size. For each alter, the participants specified age, gender, type of relationship they had with that individual (e.g., friendship, family tie), and whether they met them before or after beginning their current university degree.

#### Network satisfaction

Participants indicated whether they wished they knew more people to (1) get together with socially; (2) talk to about personal concerns; (3) ask for practical help, or whether they already knew enough people for these purposes (items adapted from Fischer [38]). Responses were examined as dichotomous binary variables; *Wish I knew more* (0); *Know enough already* (1).

#### Perceived social support

Respondents rated each alter on a 6-point Likert scale ranging from 1 (*Strongly disagree*) to 6 (*Strongly agree*), indicating whether they felt this person ‘would be there for them’ in difficult times.

#### Lived experiences of social relationships

Three open-ended questions enquired about participant social relationships prior to and after commencing university, their mental health and well-being, and the impact of the COVID-19 pandemic on their university experience and social networks. There was no limit on the length of text participants could enter to answer these questions.

### Data analyses

Participants who failed to nominate at least one alter were excluded from the analyses (n = 3), as it was not possible to ascertain whether this corresponded to a null response or missing data. All statistical analyses were conducted using R programming environment (Version 4.0.2 [39]). Though our analyses are exploratory in nature, and therefore preclude the need for power estimates, we rely on simple statistical tests requiring sample sizes of ~ 50 (e.g., chi-square tests, Pearson correlation; [40, 41]) to accommodate our relatively small sample size (*N* = 61).

The following analytical procedure was used to address our aims. First, we calculated loneliness scores and network size for each participant, as well as the mean rating of perceived social support in the network. The average age of alters, and the proportion of males and females, family members and friends, previously established/older ties and university-based/newer ties in the network were also computed. Next, we used correlation tests (e.g., Point-Biserial, Spearman) to examine associations between student loneliness and network satisfaction and social network characteristics, respectively.

Then, we explored the influence of living situation in the sample. Students living on-campus and students living off-campus were compared for loneliness scores, network characteristics and network satisfaction using t-tests and chi-square tests. Associations, measured through correlations between loneliness, network satisfaction, and network characteristics, were explored separately in these subsamples (i.e., on-campus students vs off-campus students).

Qualitative analysis of responses to the three open-ended questions was then performed using Braun and Clarke’s [42] six-stage framework for reflexive thematic analysis [43]. Data were initially coded, and themes derived by the first author (LR), while herself completing an undergraduate degree at a higher education institution in the context of the COVID-19 pandemic. LR’s own experiences of studying amid the COVID-19 pandemic may thus have influenced her approach to making sense of participants’ personal accounts.

As the present manuscript was prepared, LR had completed her degree and COVID-19 –related restrictions in the UK were significantly eased. In these changed circumstances, LR undertook the process of thematic analysis again, resulting in similar themes and overall understanding of the data. During the analysis, the analytical process and the emerging themes were discussed among authors, but data was not double coded.

## Results

### Student sample

Of the 61 participants meeting inclusion criteria for the study, 3 (2.9%) failed to nominate any alters and were therefore excluded. Sample (N = 58) characteristics are reported in Table 1. The mean loneliness score was M = 5.9 (SD = 1.9). Over half of the sample (51.7%) reported high levels of loneliness (UCLA-3 scores ≥ 6), and over three quarters (77.6%) reported a desire to socialise with more people. 56.9% indicated some dissatisfaction with the number of people they can confide in and 46.6% wished they knew more people to help them with practical things.

**Table 1:** Demographic characteristics of first-year university students included in the analyses (n = 58).

	n	%	M (SD)
***Age***			19.1 (3.1)
18–20	50	86.2	
21–23	6	10.3	
>23	2	3.5	
***Gender***			
Woman	49	84.5	
Man	8	13.8	
Non-Binary	1	1.7	
***Sexual Orientation***			
Straight/Heterosexual	46	79.3	
Bi/Pansexual	8	13.8	
Gay/Lesbian	2	3.5	
Specified: asexual	1	1.7	
PNTS	1	1.7	
***Self-reported Ethnicity***			
White	50	86.2	
Asian or Asian British	5	8.6	
Mixed	3	5.2	
***First Generation Student***			
Yes	41	70.7	
No	17	29.3	
***College of registration***			
College of Arts	18	31.0	
College of Social Sciences	15	25.9	
College of Medical, Veterinary and Life Sciences	13	22.4	
College of Science and Engineering	12	20.7	
***Diagnosis/Disability***			
None	40	68.9	
1 or more MH condition	10	17.2	
1 or more LD	2	3.5	
Comorbid MH condition & LD	2	3.5	
PNTS	4	6.9	
***Living Situation/Accommodation***			
On-campus	23	39.7	
Off-campus	35	60.3	

No Missing Data. % = Percentage; M = Mean; SD = Standard Deviation; MH = Mental Health; LD = Learning Difficulties; PNTS = Prefer Not To Say.

### Network characteristics & perceived social support in the sample

Overall, students nominated a total of 690 alters. The characteristics of participants’ ego-networks and percentage of missing data across all 690 nominated alters for each network variable are reported in Table 2. Network size ranged from 3 to 30 alters (M = 11.9, SD = 5.3).

**Table 2:** Characteristics of ego networks of first-year university students included in the analyses. (N = 58).

Alter Data (Network Characteristics)	N	M(SD)	% Missing data
**Network Size**		**11.9(5.3)**	/
Minimum network size	3		
Maximum network size	30		
**Network Gender**			2.5
÷ Woman		.65(.19)	
÷ Man		.33(.19)	
**Network Age**			8.7
Average network age		26.4(7.3)	
SD of age in Network		12.3(6.3)	
**Relationship types**			2.3
÷ Family		.28(.17)	
÷ Friends		.55(.20)	
÷ Acquaintances		.045(.09)	
**Newer vs Older ties**			2.8
÷ Older ties		.80(.25)	
÷ Newer ties		.18(.23)	
**Perceived social Support**			2.3
Perceived social support		4.9(.72)	

M = Mean. SD = Standard Deviation. % = percentage. ÷ = proportion.

### Bivariate associations: Loneliness, network satisfaction & social network characteristics

Associations between loneliness and network characteristics and network satisfaction are reported in Table 3. In the overall sample, having a larger social network was associated with lower levels of loneliness (ρ(56) = -.35, p = .008). Loneliness was not related to perceived social support nor to network composition (Table 3).

**Table 3:** Correlation matrix: Associations with loneliness in the overall sample and subsamples (on-campus vs off-campus).

**Associations with Loneliness**			
	Overall sample (N = 58)	On-campus (N = 23)	Off-campus (N = 35)
**Network characteristics**			
Network size	-.35** **(.007)**	**-.47** * **(.02)**	-.29 (.098)
÷ Older ties	-.035(.8)	.07(.8)	-.04(.8)
÷ Newer ties	.079(.6)	-.06(.8)	.12(.5)
Average support	-.19 (.2)	.23(.3)	-.40* **(.02)**
**Network Satisfaction**			
Confidants	-.27* **(.04)**	-.19(.4)	-.33 (.056)
Practical help	-.47** **(.0002)**	**-.59 (.003)**	**-.41(.02)**
Social participation	-.46** **(.0002)**	**-.43(.04)**	**-.51(.002)**

Each association is presented in the format, correlation coefficient(p-value). ÷ = proportion. Bolded correlation coefficients reflect significant associations at α = .05.

***p < .001.

**p < .01.

*p < .05..*p* < .1

Higher loneliness scores were also associated students feeling like they lacked others to ‘talk to about personal concerns’ (rpb(56) = -.28, p = .04, 95% CI [-0.50: -0.02]), get ‘help with practical things’ (rpb(56) = -.47, p = .0002, 95% CI[-0.65: -0.24]), or ‘get together socially’ (rpb(56) = -.46, p = .0002, 95% CI [-0.64: -0.23]).

### Subsample analyses by living situation

Loneliness scores (M_on-campus_ = 6.0, SD =; M_off-campus_ = 5.9, SD =; t(51.5) = -0.14, p = 0.9, 95% CI [-1.07: 0.93]), network size (t(54.9) = -1.1, p = 0.3, 95% CI [-4.15: 1.20]) and perceived social support ratings (t(47.0) = 1.3, p = 0.2, 95% CI [-0.15; 0.63]) were not significantly different for on-campus and off-campus students. Across different living situations, students did not differ either in how satisfied they were with their social networks. (Confidants, X^2^(1, 58) = 0.05, p = 0.8; Practical support X^2^(1,58) = 0.01, p = 0.9; Socialising X^2^(1, 58) = 0.8, p = 0.4).

Students who lived on-campus had, however, on average, a significantly greater proportion (M = .31, SD = .25) of university-based, newer ties compared to students living off-campus (M = .10, SD = .18; *t*(36.1) = -3.6, p = .001, 95% CI [-.34; -.09], d = .99) and lower proportion of previously established, older ties constituting their networks (M_on-campus_ = .68, SD_on-campus_ = .25; M_off-campus_ = .87, SD_off-campus_ = .21; t(41.4) = 2.9, p = .006, 95% CI [.06; .31], d = .79).

#### Network characteristics

In students who lived off-campus, higher average ratings of social support across alters were associated with reduced loneliness (ρ(33) = -.40, p = .02) (Table 3). For on-campus students, however, larger networks were significantly related to lower loneliness levels (ρ(21) = -.47, p = .02). The proportion of previously established or university-based social ties was not related to loneliness in either group (Table 3).

#### Network satisfaction

The negative association between loneliness and satisfaction with the number of individuals available for practical help was significant in both the on-campus (rpb(21) = -.59, p = .003, 95% CI [-0.81: -0.23]) and off-campus group (rpb(33) = -.41, p = .02, 95% CI [-0.65: -0.083]). Similarly, in terms of socialising, network satisfaction was linked to loneliness in both on-campus (rpb(21) = -.43, p = .04, 95%CI [-0.71: -0.017]) and off-campus (rpb (33) = -.51, p = .002 95%CI [-0.72: -0.22]) students. Satisfaction with the number of confidants was neither associated with loneliness in students living on-campus (rpb(21) = -.19, p = .39, 95% CI [- 0.56: 0.24]) nor among students living off-campus (rpb (33) = -.33, p = .056, 95% CI [-0.6: 0.009]).

### Qualitative results

Fifty-seven respondents (94.3%) provided responses to the open-ended questions. Three overarching and interrelated themes were identified in the data, revealing how the impact of the COVID-19 pandemic on students’ social networks during their transition to university closely intertwined with their academic experience and well-being.

#### Social networks in the COVID cohort: The continued importance of pre-existing ties

Students’ networks after the first months at university seemed still predominantly comprised of their well-established, long-standing pre-university relationships, whilst the extent to which new university-based ties were viewed as important to their networks was somewhat limited. Students wrote about having close and supportive ties with school/childhood friends and family, as well as having had many people for socialising before starting university. Even after the transition to university, these long-standing ties were still considered an important part of students’ networks. Students gave examples of the ways in which they had been keeping in touch since they started university (e.g., social media and online chatting, telephone calls) to demonstrate their continued contact and persisting connection with these older ties. Others stated this more explicitly:

*“Before moving to university I had really close ties with multiple people and since moving I have kept in good contact with all these past relationships, i.e. boyfriend, best friends and family members” (P31)*

*“My social relations between my family and I, my friends and I, have been and carry on to be strong throughout and before Uni” (P59)*

On the other hand, in most students’ responses, university-based ties were either described as non-existent or as weak. Whilst a few students reported some positive new relationships with other students, often from living in shared halls of residence, most students reported having “made no new relationships since coming to university” (P25). University peers were not seen as principal sources of support or as people to “depend on so far” (P53) nor did students feel they had yet established strong relationships with other students that could be truly qualified as ‘friendships’. Rather, university-based ties were often explicitly compared to the previously established, long-standing social ties (constituting the ‘real’ social network)

*“I feel that I have developed very few social relationships since coming to university; with some new acquaintances but no new friendships” (P51)*

*“Before university all of my social relationships were relatively solid, dependable and positive. I have not yet made any new social relationships since coming to university but my preexisting ones are mostly the same” (P36)*

#### Impact of COVID-19: Impeding new connections & changing the context of pre-existing relationships

COVID-19 crucially limited students’ opportunities for integrating within university-based social circles and developing meaningful new connections with other students in the transition to university. Although students’ circumstances varied (studying from remote off-campus or closer/on-campus locations), most discussed how the inaccessibility/closure of traditional spaces for face-to-face student interaction both in an academic (e.g., lecture halls) and leisure (e.g., student unions; pubs) capacity, resulted in limited opportunities to develop university-based social networks. Students believed that if they had been attending “social events” (P06) or “going out more and going to classes in person” they would have “met more people” and “made a lot more friendships.” (P20).

Even when they wrote about living with other students, respondents felt limited by the lack of alternative options and opportunities for socialising and expanding their social circles at university beyond those they lived with.

*“Although I am friends with my flat mates I wish I knew more people than I do” (P52)*

*“I also felt that the restrictions meant we were forced to only socialise with the people in our flat meaning I didn’t make as many friendships as I though I might.” (P61)*

Multiple and varied opportunities for interacting face-to-face with other students were therefore seen as crucial for the development of new friendships whilst having to interact online seemed to limit meaningful interactions and in turn, the development of closer bonds with other students and stronger university-based ties.

*“There are some people I still haven’t been able to meet in person and so I think this can cause the relationships to not be as close as they would be without covid” (P32)*

Students also reflected on the impact of social-distancing measures and COVID-19 related travel restrictions on their relationships with members of their pre-existing networks. This included changes in the frequency and type of activities they could do with the important and long-standing members of their social network (family, friends) which seemed to create concerns about relationships being ‘disrupted’ or having “drifted” (P30), because they were unable to “hang out in the same way or very often” (P32) or to “get to see them as often as [they] would want to anymore” (P08). Thus, whilst pre-existing ties were still predominant in students’ networks after the transition to university (cf., theme 1), it nonetheless seemed that not all had been maintained.

Students mainly discussed the negative impact that the COVID-19 pandemic had had on their ability to establish new social ties at university, and on the difficulties it had posed in maintaining the whole array of their previously established social networks. However, some recognised that social ties within a specific social bubble, often defined by their household (e.g., family home, specific flat in a student accommodation) could be reinforced in the pandemic context. Indeed, in these cases, restrictions on being able to socialise out-with this bubble was reported by some students as having led to a positive increase in the amount of time spent with those within their ‘household’.

*“Since there is a lockdown, we find ourselves spending more time with each other every single day. We watch movies almost every night and study together (P23)*

*“I have a good relationship with my family–- and we spend more time together recently due to COVID” (P12)*

*“I have become closer to people in my accommodation than if there hadn’t have been covid–- impacted heavily due to isolation and a lack of nightlife” (P26)*

#### Social ties and mental health in the transition to university among the COVID cohort

Students’ expectations for the first year at university were that of a fun, exciting time for meeting other students, integrating into social circles and settling into their life at university. This seemed to be seen as defining a “fresher’s experience” (P37) and to have indeed been greatly disrupted in the context of COVID-19. Students’ accounts thus revealed strong feelings of frustration and a sense of having been deprived of some integral aspects of their student experience, which ought to have characterised their transition to university.

*“It feels very unfair that I (along with all other freshers this year) have missed out on what should have been such a fun year, and a time to meet so many new people”. (P16)*

*“[My fresher’s experience has been affected] In every way. Not being on campus a lot, if ever. Not being able to meet people on my course. Not being able to go out to bars, clubs or other student’s flats. It has single handedly ruined many people’s first year at university which is arguably the most important social year.” (P41)*

Students explicitly linked a decrease in socialising during the transition to university to a decrease in well-being and an increase in negative experiences and mental states. Students notably reported that they felt ‘lonely’, ‘isolated’, ‘sad’ and ‘anxious’ as a result of the pandemic stopping them both from spending time with their friends and meeting new people (cf. theme 2). Social distancing measures also seemed to evoke feelings of being ‘trapped’ and ‘stuck’ which were described by students in relation to both an emotional (e.g., “been stuck inside my own head” (P26)) and physical state (e.g., “being stuck inside the house” P10).

Students discussed how the context of the pandemic had led them to reflect on their social relationships, eliciting feelings of empowerment and gratitude for the support of their close ones, but also a sense of loss and loneliness.

Emotional difficulties seemed both linked to and compounded by changes in students’ social networks in the context of the pandemic and the transition to university. This, in turn, could create a sense of exhaustion, anxiety and lack of energy, leading them to further withdraw from engaging with some of their existing ties and from developing new connections.

*“For the first few months I stayed positive but now the pandemic is starting to take it’s toll on my moods. Losing touch with most people because I am not in the mood to keep up with them” (P17)*

*“COVID has allowed me to think a lot about the importance of certain friendships so in a way now, I redimensioned my view on friendships. At the same time though, I feel more at ease on my own than I ever did. Although this is empowering and positive, it is also really negative as I don’t feel like seeing my friends a lot, nor developing new friendships. I feel isolated” (P08)*

## Discussion

The current study adopted a mixed-methods approach to assess the links between social networks and loneliness in the COVID cohort of undergraduate students. Via exploratory quantitative analysis, we identified that having a larger social network, and higher satisfaction with that network was associated with reduced loneliness amongst our sample. Loneliness was associated with student satisfaction with the number of people they can confide in, rely on for practical help, and get together socially, which have all been identified as important functions of social networks for supporting adjustment in the transition to university [15, 44]. Living on or off-campus was not associated with loneliness, network size, perceived social support or satisfaction with social networks in our sample. However, the networks of those living on-campus differed to those living off-campus in that those on-campus had a greater proportion of new social ties. Associations with loneliness also differed between the two sub-groups, although, given the exploratory nature of these analyses, these findings should be interpreted with caution.

Overall, quantitative analyses suggested that loneliness was associated with how socially integrated students are (i.e., network size) and how adequately their needs are met by their social networks. Qualitative analyses identified three interrelated themes, which demonstrated the impact of the COVID-19 pandemic on students’ social networks and well-being. Qualitative data contextualises quantitative findings and adds rich insights into the lives of young people beginning their undergraduate studies in the context of COVID-19 and associated restrictions, and how these have impacted on their ability to forge meaningful social relationships.

Times of transition are linked to increased vulnerability in experiencing loneliness [45, 46]. While the evidence indicates that loneliness increased in young people during the COVID-19 pandemic [47], and that students were particularly at risk of loneliness [48], few previous studies examined first-year students making the transition to undergraduate study within the context of the pandemic and associated lockdowns. The current study harnessed both quantitative and qualitative data to gain a comprehensive understanding of the experience of the COVID cohort. The qualitative data revealed that pre-university social ties were a vital and significant component of students’ social landscape, and that most reported making no new relationships since starting university. This is in contrast to the findings which suggest that starting university has traditionally been a time in which new social connections are forged [49, 50].

Previous research has noted, for example, that in the first days of attending university, social ties at home provide a buffering effect against the stress of transition, but as students develop new social connections at university, these become their primary source of social support [51]. Therefore, while in a pre-pandemic setting, university-based connections would become key sources of support, those in the COVID-cohort were unable to transition from having primarily home-based social support, to university-based social support due to restricted social opportunities, as reflected by our qualitative findings. This places those in the COVID-cohort at risk of reduced integration into university life, and increased risk of withdrawal from studies [52]. Therefore, universities should aim to increase social opportunities for this cohort.

Previous research has asserted that social network interventions, which aim to provide contact opportunities among undergraduate students, may provide an initial short-term boost to social networks, such as greater rate of reported friendships, common friends, and mixed gender friends [53]. Such interventions have a long-term benefit in the creation and maintenance of more complex social networks [53]. Social-distancing restrictions have now eased, and universities have continued adopting hybrid teaching models [54]. Social network interventions may thus provide a useful avenue to bolster the social lives of students in the COVID-cohort, particularly as some evidence suggests that students’ social network struggled to recover from reductions in size after the period of lockdown [26]. As noted above, such interventions may not only be useful for improving social connection and reducing loneliness [47] but may also help improve educational outcomes of students [52].

In our sample, larger network size was associated with reduced loneliness, and on-campus and off-campus students didn’t differ in relation to loneliness, network size or perceived social support. Although the on-campus students reported more new social ties than those living off-campus, our qualitative findings suggest that these relationships were perhaps superficial in nature, and that lockdown restrictions limited opportunities to develop more meaningful ones. Similar experiences were reported in a qualitative study conducted by Lippke and colleagues [55] in the midst of a lockdown, where students contrasted their connections with other university students against their “real friends” and discussed their lack of deep friendships and meaningful connections at university. These qualitative findings further highlight the importance of considering students’ satisfaction with their social connections, which is also suggested by quantitative associations in our overall sample. In our exploratory subsample analyses, we observed an association between greater social network size and lower loneliness among students who lived on-campus, which was not observed among students living off-campus. On the other hand, loneliness was associated with perceived social support in the off-campus group, which was not replicated in the on-campus group. In other words, for students who lived on-campus among their university peers, their level of social integration (size of their social networks) was related to feelings of loneliness, whilst for students who lived away from campus, the supportiveness of their social circle showed a stronger association.

It may be that students living off-campus were afforded a more familiar social environment to buffer the transition into university life, which is a known stressor and risk factor for loneliness [45, 46]. This may protect against loneliness, even if opportunities for social connection are less plentiful. Indeed, previous research has noted that the transition to university may be particularly difficult for those living on-campus, when social ties with friends from school tend to reduce in satisfaction, supportiveness, and quality [13, 15, 49], while those who study off-campus and remained at home are more likely to see these pre-established friendships and social supports maintained [56].

In our qualitative findings, students across different living situations still reflected on the detrimental impact of COVID-19 restrictions on opportunities for meeting other students due to often working remotely and attending fewer social events. It has been argued that the negative feelings associated with loneliness can serve an adaptive purpose of motivating a need to seek social contact and form social connections [57, 58]. Social distancing measures impeded the ability to form such connections, and thus may have increased the psychological distress of students in the COVID-cohort [59, 60]. Additional research examining loneliness in the UK during the COVID-19 pandemic identified that being a student and being aged 16–24 years were risk factors for increased loneliness, while having more close friends, and more friends of a similar age was likely to serve as a protective factor against loneliness [61]. This, therefore, burdened the COVID-cohort with uniquely risky set of factors for increased loneliness.

The lack of opportunity to socialise and form new, and supportive ties among those starting university during the pandemic and associated restrictions may be particularly detrimental to the wellbeing of the COVID cohort. The National Union of Students (NUS) found that 52% of students studying in the UK in the academic term 2020–2021, reported that their mental health was worse than compared to before the pandemic [62]. Furthermore, our qualitative findings identified that students felt an additional sense of frustration at having been deprived of essential social aspects of the student experience. The disconnect between anticipated social opportunities, and their new social realities may have further thwarted attempts to form meaningful social connection to mitigate loneliness and indeed thrive in the transition to university [63].

### Limitations and future research

Our qualitative responses were collected as open-ended text responses embedded within the larger survey. Therefore, undergraduates may have had additional experiences or understandings of the impact of COVID-19 that they did not share within the context of the survey.

The current quantitative findings were based on a relatively small sample of students attending a single university (*n* = 58), which may limit their generalisability to other settings, such as universities outside of large urban areas. In light of the exploratory nature of our study, findings should be interpreted with caution and require further investigation to increase confidence in the observed relationships between social network variables and loneliness. We relied on simple statistical measures, such as correlations and bivariate associations, in order to overcome our small sample. We maintained a significance level of p < 0.05 across our tests, reflecting both our sample size and exploratory nature of the analyses. Future studies would benefit from replicating our analyses with larger sample sizes, which would promote greater generalisability, and allow for multiple tests (e.g., repeated correlation tests) to be taken into account.

In the present study, we chose to focus on the COVID cohort, due to the expected risk for high levels of loneliness among these students who began their studies at the height of the pandemic. However, our findings may not be applicable to first year students who experienced the transition to university in the context of different phases of the pandemic and post-pandemic. Further research may sample students from these cohorts who started their undergraduate studies under different levels of restrictions on social contact (e.g., none, social distancing, full lockdown) and experienced different modes of teaching delivery (e.g., remote, hybrid) as a result [19, 20]. Examining which aspects of social relationships and networks are associated with loneliness and how students’ living situations influence these associations among cohorts of students who experience different sets of circumstances and opportunities surrounding their transition to university may help better understand how to support first year undergraduates at the start of their studies. This is of particular interest in the context of hybrid models of teaching being widely maintained by universities even as social restrictions were eased and lifted [54].

Consistent with our aim to assess the relationship between social networks and loneliness rather than establish causality, we used cross-sectional methods, and therefore could not account for pre-existing levels of loneliness among surveyed students. Future longitudinal models would help to address this. Notwithstanding, our quantitative results are enriched and contextualised by valuable qualitative findings, offering more detailed insights into students’ experiences and their understanding of the roles of social relationships (and their disruption) in the transition to higher education.

## Conclusion

The current findings contribute to understanding the role of social networks in facilitating the transition to higher education and promoting first-year students’ mental health and wellbeing during adjustment to university life. We harnessed both qualitative and quantitative data to explore associations between the characteristics of students’ social networks and loneliness, in a sample of students whose opportunities for social interaction were greatly limited. Our results suggest that, in the transition to university, it is important for students to have both plentiful, varied opportunities to meet others (formally and informally) and to form meaningful connections, in order to develop social networks which can meet their social needs according to their individual circumstances (e.g., living situation). In the context of universities continuing to implement hybrid teaching models [54], these findings highlight avenues for institutions to investigate further when implementing initiatives to support students developing adequate social connections in the transition to higher education.
